# Pollinator foraging flexibility mediates rapid plant-pollinator network restoration in semi-natural grasslands

**DOI:** 10.1038/s41598-019-51912-4

**Published:** 2019-10-29

**Authors:** Norbertas Noreika, Ignasi Bartomeus, Marie Winsa, Riccardo Bommarco, Erik Öckinger

**Affiliations:** 1 Swedish University of Agricultural Sciences, Department of Ecology, PO Box 7044, SE-75007 Uppsala, Sweden; 2 University of Tartu, Institute of Ecology and Earth Sciences, Lai 40, EE-51005 Tartu, Estonia; 3 Estación Biológica de Doñana (EBD-CSIC), Departamento Ecología Integrativa, Avda. Américo Vespucio s ⁄n, Isla de la Cartuja, E-41092 Sevilla, Spain

**Keywords:** Grassland ecology, Ecological networks, Community ecology, Restoration ecology

## Abstract

We examined how plant-pollinator interactions were affected by time since habitat restoration and landscape connectivity by comparing plant-pollinator networks in restored, abandoned and continuously grazed semi-natural pastures in south-central Sweden. We measured richness of flowering plants and pollinators, and local plant-pollinator network characteristics including species composition as well as the number and identity of interactions, allowing a deeper understanding of species and interaction beta diversity. Pollinator richness and abundance were highest in restored grasslands. They successfully resembled continuously grazed grasslands. However, the turnover of interactions was extremely high among pasture categories (0.99) mainly due to high turnover of plant (0.74) and pollinator species (0.81). Among co-occurring plant and pollinator species, the turnover of interactions (0.66) was attributable mainly to differences in the number of links and to a lesser extent to species true rewiring (~0.17). Connectivity and time since restoration had no effect on the measured network properties. We show that plant-pollinator interactions can be rapidly restored even in relatively isolated grasslands. This is partly due to flexibility of most pollinators to establish interactions with the available flowering plants and relatively high species interaction rewiring, indicating that pollinators behavioural plasticity allow them to shift diets to adapt to new situations.

## Introduction

The overarching goal of ecological restoration is to assist the recovery of a degraded ecosystem such that it sustains species communities and ecosystem functions without further interventions^1^. The success of ecosystem restoration is often evaluated based on the recovery of community composition^2^, while the recovery of ecological functions and processes is only assumed^1^. Most ecosystem functions are driven by different interactions among species^3^, including plant-pollinator mutualistic relationships. Hence, investigating not only plant or pollinator composition, but full plant-pollinator interaction networks in restored habitat fragments is a promising approach to evaluate if community composition and ecosystem functioning have recovered after ecosystem restoration^4–7^.

The structure of plant-pollinator networks is determined by several factors^8^. For instance, two species interact only if they co-occur in space and time, which results from their distribution and abundance. Further, only those co-occurring species which have matching morphological and physiological traits will interact. Also, species richness and abundance determine competition among pollinators and modulate their foraging behaviour which, in turn, can reshuffle interactions among species^9,10^. Understanding how and why plant-pollinator networks vary across time and space is still an open question^7,11,12^. Assessments of spatiotemporal changes in plant-pollinator networks would increase our understanding of the processes shaping network (re-) assembly. This could provide information on how to more efficiently restore ecosystem functions^13,14^.

The first step to assess the effectiveness of restoration actions in restoring plant-pollinator networks is to compare the species composition in restored sites to that in intact reference sites or abandoned non-restored sites by means of, for example, species beta diversity indices^15^. Similarly, beta diversity indices can be further used to reveal causes for variation among species interaction networks^16–19^. Differences in species interaction link structure among local networks can arise from (i) species composition differences among sites (species beta diversity), or (ii) turnover of species interaction links among co-occurring species (interaction beta diversity)^11,20^. Such turnover is often being referred to as “re-wiring”, reflecting the capacity of pollinators to re-shape the link structure depending on the context. However, most used metrics are actually not able to distinguish pollinators true rewiring from pollinators simply visiting a narrower subset of plant species. To that end, beta diversity metrics for both species and interactions can be further partitioned to explore whether beta diversity is driven by real turnover of species and links, or by differences in the numbers of species and/or links^21^. Overall, this framework allow us to separate if observed differences among plant-pollinator networks are mainly explained by species composition, or by rewiring of interactions.

Beyond different management regime comparisons, plant-pollinator restoration success can be also evaluated by comparing the restored network with the potential regional network. The metaweb concept, which consists of all possible links of interacting species within a study region^22^, is a useful tool for revealing possible mechanisms of network formation^11,17^. Thus, it could be used to infer how efficient the restoration is in terms of realized networks in the region and also provide knowledge on the causes of such realization^11,17^. Semi-natural grasslands are key ecosystems for investigating how plant-pollinator network recover after ecological restoration. They have high plant species richness^23,24^ due to extensive grazing or hay-making over centuries^25^. This rich plant community combined with an abundance of structures that can provide suitable nest sites, semi-natural grasslands are important source habitats for pollinators in agricultural landscape^26,27^. Semi-natural grasslands represent one of the highly threatened habitat types in Northern Europe, since many of them have been either abandoned or converted into arable land or forest^28^. To counteract the loss of this biodiversity-rich habitat, degraded semi-natural grasslands are now being restored^29^ often with positive effects on both plants and insects^30,31^. However, plants and pollinators can show contrasting responses to grassland restoration^32^, with potential consequences for species interactions, but plant-pollinator interaction responses to habitat restoration have so far only received limited attention (but see^2,5,33^).

Habitat connectivity and the time since restoration are key variables that could affect the re-assembly of interaction networks. We have previously found that the restoration success of Swedish grassland plant and pollinator communities and their associations is highly dependent on site connectivity^34,35^ and on the dispersal ability of both pollinators and plants^32,34^. In addition, plant trait composition in restored sites changed gradually with increasing time since restoration^32^. Thus, we are interested on how connectivity and time since restoration modulates the restoration process of plant-pollinator networks.

In order to reveal how plant-pollinator networks recover after restoration of semi-natural grasslands, we decomposed beta diversity of pairwise interactions among sites within three different pasture categories (a space-for-time study design): abandoned, restored and continuously grazed. We further investigated how these networks were affected by connectivity to continuously grazed pastures as well as time since restoration. We hypothesized that: (1) plant and pollinator species diversity is higher in continuously grazed than in abandoned and restored; (2) interaction and species beta diversity among restored and among continuously grazed sites are low and similar if restoration is successful, i.e. a complete restoration not only restores species composition but also the interaction networks; (3) interactions and species beta-diversity in restored vs. continuously grazed sites decrease with increased connectivity to other grasslands and time since restoration; (4) within abandoned pastures, we expect that a dominance of a few, but similar species will result in low network turnover of species and links, i.e. low species and interaction beta diversity; (5) a higher proportion of interactions from the regional metaweb is realized after successful restoration.

## Methods

### Study system

**Figure 1:**
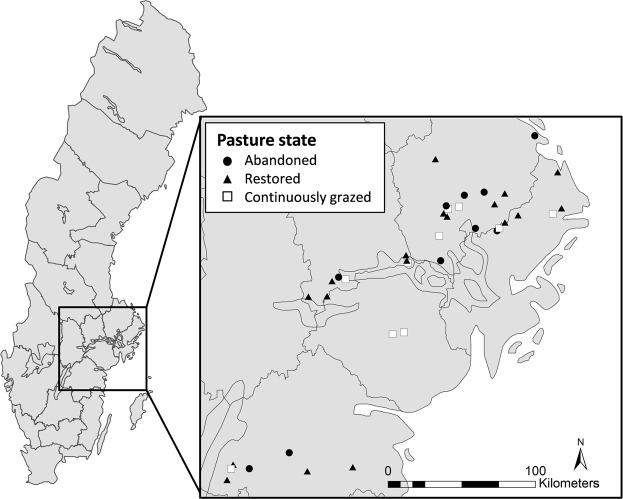
Map of the study area in south-central Sweden, including 38 semi-natural pastures of different management states (abandoned, restored and continuously grazed) in landscapes with contrasting connectivity to continuously grazed pastures. Surveys of flowering plants, pollinators and their interactions were conducted in each pasture on five occasions in the period May to July 2012. The map was created using ArcGIS 10.3, based on background maps from Lantmäteriet, the Swedish National Land Survey, under license i2014/764.

To study plant-pollinator interactions in relation to pasture management history and connectivity, we used previously established site system^32,35^. In the current study we collected plant-pollinator network data which is new and complementary to the plant and pollinator community data used in our previous studies. We explored 38 semi-natural pastures in south central Sweden (in the counties of Västmanland, Uppland, Stockholm, Södermanland and Östergötland). Using a space-for-time substitution, we included 10 abandoned, 18 restored and 10 continuously grazed semi-natural pastures of dry or mesic type, where the abandoned pastures represent the state before restoration, and the continuously grazed pastures represent the reference state (Fig. 1)^32,35^. The pastures were selected along a connectivity gradient to continuously grazed semi-natural grasslands in the surrounding landscapes, and restored pastures ranged in time since restoration from 2–16 years.

To select the abandoned and continuously grazed pastures we used a national Swedish geographical database of semi-natural grasslands (http://www.jordbruksverket.se/tuva). The selection of continuously grazed pastures was based on habitat type, such that the pastures displayed similar vegetation structure. Restored pastures were located and selected with information from The Uppland foundation, the County Administrative Boards and the municipalities in the regions, on pastures for which land-owners had received economical compensation to restore from an abandoned state. Restoration actions consisted of removal of encroaching shrubs and young trees, and by re-establishment of grazing livestock (cattle or sheep).

For each pasture, we calculated connectivity to all other semi-natural grasslands (meadows and pastures) within a 5 km radius, using the index described by^36^:

$$C{I}_{i}=\varSigma exp(\mbox{--}\alpha {d}_{ij}){A}_{j}^{b}$$

where *A*_*j*_ is the area of the neighbouring fragment *j* (in hectares) and *d*_*ij*_ is the distance (in km, centre to centre) from the focal fragment *i* to the neighbouring fragment *j*, *α* is a species-specific parameter describing a species’ dispersal ability, and *b* is a parameter that describes the scaling of emigration as a function of patch area. Since we applied the connectivity index to an entire community with several taxa, we set the parameter *α* to 1 (corresponding to an average dispersal distance of 1 km) and *b* to 0.5. Values of *b* close to 0.5 has been observed for several insect species. The rank order of pasture connectivity is not sensitive to the value of these parameters^37^.

Pasture area ranged from 1 to 13 ha, with a mean area for abandoned pastures of 2.6 ha (SE = 0.2, median = 2.7), for restored pastures 3.8 ha (SE = 0.7, median = 3.3) and for continuously grazed pastures 2.8 ha (SE = 0.5, median 2.3). Connectivity ranged from 0.1 to 26.6. The mean connectivity for abandoned pastures were 8.7 (SE = 2.2, median = 7.3), 8.3 (SE = 1.8, median = 5.6) for restored pastures and 7.6 (SE = 2.3, median = 6.9) for continuously grazed pastures.

### Pollinator and flower visitation surveys

Bees and hoverflies were surveyed during May-July in 2012. Each pasture was visited five times during this period. In each pasture, all pollinators, both those observed flying and those observed to visit flowers were recorded along four 50 m transects per site (i.e. in total 200 meter transect per site and visit). Transects were two meter wide, and were placed such that they would capture the habitat variation within each site. Pollinators were collected and identified in the lab, and flowers that were visited by pollinators were determined to species in the field. Surveys were made in the morning and in the afternoon every second time during standardized weather conditions regarding temperature (≥16 °C), max. 50% cloud cover, no precipitation and low wind (≤4 m/s).

### Floral resources

To quantify floral resources in the pastures, we counted the number of solitary flowers or inflorescences per flowering plant in three plots of 1 × 1 meter per pollinator transect of 50 meters (i.e. in total 12 plots per site and visit). The plots were placed at regular distances along the pollinator transects (5, 25 and 45 m).

### Statistical analyses

Flower visitation by honey bees (*Apis mellifera* L) accounted for only 4% of the visits recorded, and were excluded from all analyses as our focus and interest concentrated on non-managed bees. All statistical analyses were performed in R software, version 3.3.3^38^.

### Network characteristics

The number of plant and pollinator species and number of links per species were explored for each site’s network using the ‘bipartite’ package^39^. All recorded plant species were included in the beta diversity analyses: plants recorded from 1 × 1 m investigation plots, and plants visited by pollinators but absent within the 1 × 1 m plots. The latter plant species were assumed to be rare and were set to an abundance value of 1.

### Calculation of beta diversity

Differences in network structure among pastures are manifested in beta diversity of interactions. First, beta diversity metrics of species (based on binary and quantitative data) were calculated by using *vegdist* (Ruzicka index on quantitative data, see below) and *betadiver* (Colwell and Coddington index on binary data, see below) functions in ‘vegan’ package^40^. Interaction beta diversity (See Table 1 for overview) was obtained from a pairwise comparison between networks using both binary (based on presence/absence of links) and quantitative (based on frequencies of interactions) data in a modified quantitative version of the ‘betalink’ package^11^, modified in^41^). Hence, we calculated total network beta diversity of species (pollinators: U, plants: L) and interactions (WN) to assess dissimilarities in local network structures, and also beta diversity of interactions between shared species (OS) to assess dissimilarities in interactions between co-occurring species among local networks. Binary networks comprises the structure of interactions in terms of presence/absence of links between species^42^. Binary diversity calculations were based on the^43^ measure (Eq. 1):

1 $${\beta }_{CC}=(b+c)/(a+b+c)$$

where *a* is the number of species common to both sites, *b* is the number of species exclusive to the first site, and *c* is the number of species exclusive to the second site^21,44^.

**Table 1:** Beta diversity measures used in pairwise comparison of networks, including measures of dissimilarities in both species and interactions. The beta diversity measures can be further partitioned to explore to what extent differences in number of species/links, or differences in turnover of species/links contribute to differences in beta diversity. This is here referred to as ‘second decomposition’.

Beta diversity measures	Description	Second decomposition
L	Compositional turnover in lower trophic level (i.e. plants)	L3 (plant species turnover) + Lrich (plant species richness differences)
U	Compositional turnover in higher trophic level (i.e. pollinators)	U3 (pollinator species turnover) + Urich (pollinator species richness differences)
WN	Dissimilarity of interactions in two whole networks	WN3 (link turnover) + WNrich (differences in number of links)
OS	Dissimilarity of interactions between shared species in two whole networks	OS3 (link turnover among shared species) + OSrich (differences in number of links among shared species)

Quantitative networks include the weight of interactions in terms of interaction frequencies that describe the strengths of interactions^42^. Quantitative diversity calculations were based on the Ruzicka (=quantitative Jaccard index) distance coefficient (Eq. 2):

2 $${\beta }_{Ruz}(B+C)/(A+B+C)$$

where *A* is the sum of intersections (or minima) of species abundances at two sites, *B* is the sum at site 1 minus *A*, and *C* is the sum at site 2 minus *A*^45^.

Beta diversity, both of species and interactions, is determined by species or link replacement (true turnover) and by species richness or number of link differences (species or link loss or gain). We therefore partitioned the beta diversities of species and interactions into these two components following equations in^21^ (Table 1) (Eq. 3):

3 $${\beta }_{CC}={\beta }_{-3}+{\beta }_{Rich}$$

*β*_*CC*_ is the same as in Eq. 1, *β*_*Rich*_. is defined below (Eq. 5). Turnover of species or links (*β*_*−3*_) was calculated by dividing minimum number of substitutions of species (or links) between two sites by total number of recorded species (or links), i.e. (Eq. 4):

4 $${\beta }_{-3}=2{\rm{x}}(({\rm{\min }}({\rm{b}},{\rm{c}})/({\rm{a}}+{\rm{b}}+{\rm{c}}))$$

Finally, total species richness (or number of link) difference between two sites was calculated following the formula (Eq. 5):

5 $${\beta }_{Rich}=|({\rm{b}}-{\rm{c}})|/({\rm{a}}+{\rm{b}}+{\rm{c}})$$

See^21^ for details.

When analysing interaction network data, it should be noted that the sampling of networks is inevitably biased towards capturing the assembly and links of abundant species, since rare species and interactions have a lower detection probability^46,47^. Quantitative analyses of networks are generally less sensitive to sampling effort than analyses of binary data^48^. In our case, the two approaches displayed similar patterns in network beta diversity. We therefore report the results from the quantitative networks in the main text, and results from binary analyses in Supplementary information, Table S1.

### Beta-diversity comparison between local networks and the metaweb

Beta diversity of species and interactions among networks can be calculated at different scales. Here, in addition to the comparison between local networks, we also compared each local network to the aggregated network of all observed interactions across all investigated sites, referred to as the ‘metaweb’^11,22^. The metaweb provides information about the regional species pool and the interactions that co-occurring species display in the entire system. The comparison between local networks and the metaweb provides information on local resource selectivity. For instance, if local selectivity is high, beta diversity comes close to 1, if low, beta diversity comes close to zero^11^. Since the metaweb gives information on the regional species pool, an increasing part of the metaweb is expected to be realized within local networks in well-connected pastures with high connectivity to continuously grazed grasslands in the landscape.

### Impact of landscape, management and time on interaction networks

First, by using generalized linear models (GLM) we assessed how species number and links per species within networks related to grassland management (i.e. pasture category) and grassland connectivity in the landscape. The data on the number of species within the networks displayed overdispersion. Therefore we applied negative binomial generalized linear models (glm.nb) using the package ‘MASS’^49^. For number of links per species we applied GLMs with a ‘Gamma’ distribution. We ran likelihood ratio tests comparing the models with both ‘Connectivity’ and ‘Pasture category’ included, to models where one of the variables was excluded. For networks in restored pastures we ran GLMs to test the effect of ‘Time since restoration’ and ‘Connectivity’ on species number and number of links.

**Figure 2:**
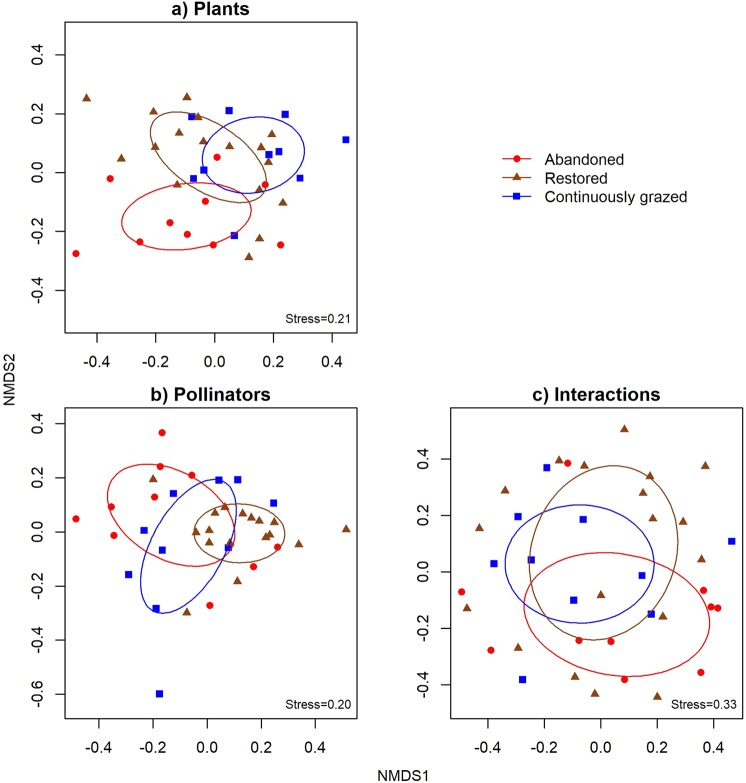
NMDS visualizing beta diversity (**a**) plant composition, (**b**) pollinator composition, and (**d**) interactions within plant-pollinator networks in the three pasture categories. One outlier was removed from all visualizations.

Second, based on the distance matrices from the pairwise comparison of local networks, we tested if beta diversity of species and interactions differed among pasture categories using Permutational Analysis of Variance (PERMANOVA^50^; using the function ‘adonis’ in the ‘vegan’ package^40^). To visualize differences and spread in beta diversity measures among pasture categories we used nonparametric multidimensional scaling (NMDS), using the package ‘vegan’^40^. Each point in the plot (see Fig. 2) corresponds to the beta diversity of species or interactions for one network compared with all other networks (based on the distance matrix). The spread of points describes differences in beta diversity of species or interaction compositions within each pasture category, and the ellipses describe the average differences in beta diversity of species or interactions among pasture categories. When plotting the beta diversity networks using NMDS, one outlier obscured the patterns in plant and pollinator beta diversity, making it difficult to assess how plant and pollinator beta diversity differed among pasture categories. The deviating network, including only one plant-pollinator interaction, was observed in a relatively recently restored pasture (4 years), where flower abundance was low and the vegetation had not recovered after clearing of trees and shrubs. This network was removed only from NMDS visualization, but it was maintained in the PERMANOVA analysis.

Finally, from the comparison between local networks and the metaweb, we assessed the effect of pasture category, connectivity and time since restoration on species composition and on interactions among shared species. Beta diversity measures ranged between 0 (complete overlap) and 1 (no overlap). In GLM models we therefore applied a beta distribution using the package ‘glmmadmb’^51^. The beta regression applied can only handle values between zero and one and not the integer values of 0 and 1 (i.e. 0 < x < 1). Therefore, we assigned the zeros (N = 4) a value of 0.0000001. Since we were only interested in the relative differences between sites and not in the specific values, adding this small number does not influence the interpretation of the results.

With increasing connectivity between focal pastures one would expect decreasing network beta diversity as a result of more similar species composition with decreasing distance between sites^18,52^. However, because we only measured connectivity to continuously grazed grasslands in the landscape surrounding each focal pasture and not connectivity between pairs of focal sites, it was only possible to assess the effects of connectivity on local network similarity to the metaweb.

## Results

### Overall species richness and abundance

When all flowering plants were considered, the average number of flowering plant species per site was 26.4 (SE = ±1.4), and the average flower abundance (solitary flowers or inflorescences) per square meter per visit, was 18.3 (SE = ±2.15) flowering units. Neither the number of plant species nor flower abundance differed among pasture categories (ANOVA: Plant species number: F_2,35_ = 1.4, P = 0.26, flower abundance: F_2,35_ = 1.56, P = 0.23, Supplementary information, Table S1).

**Figure 3:**
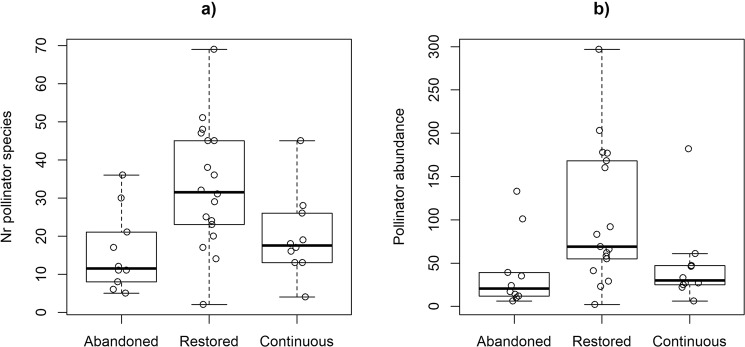
(**a**) Number of pollinator species and (**b**) abundance of pollinator per site (N = 38) and pasture category (one outlier restored site with 759 individuals is not plotted). Species numbers correspond to all observed pollinator species and individuals within the pastures, not only the ones included in local plant-pollinator networks. Raw data points for each pasture are shown in the background of the boxplots.

When all observed pollinators were accounted for, i.e. both species caught flying and species observed to visit flowers, a total of 104 species of hoverflies and 80 bee species were recorded in the surveys (Table S2). Out of these, 44 species of hoverflies and 52 bee species were recorded to visit a total of 83 flowering plant species. In total we observed 153 flowering plant species. The average number of observed pollinator species (bees and hoverflies combined) per site was 24.6 (SE = ±2.4) (Restored = 32.5 ± 3.7, Continuous = 19.6 ± 3.5, Abandoned = 15.5 ± 3.2), and differed among pasture categories (F_2,35_ = 6.18, P = 0.005, Fig. 3a). A post hoc Tukey test revealed that restored pastures had a higher pollinator species richness than both abandoned (Tukey HSD test: P = 0.01) and continuously grazed pastures (P = 0.05). The patterns were similar when hoverflies and bees were analysed separately (ANOVA, Hoverflies: F_2,35_ = 3.35, P = 0.047, Bees: F_2,35_ = 8.0, P = 0.001, Supplementary information, Fig. S1 and Table S1). The average number of observed pollinator individuals per site was 85 (±20.1) (Restored = 133.22 ± 38.45, Continuous = 46.8 ± 15.83, Abandoned = 37.3 ± 12.85) and tended also to differ among pasture categories (ANOVA: F_2,35_ = 2.83, P = 0.07), with the highest abundance again in restored pastures (Fig. 3b). However, the pollinator abundance was very variable within pasture categories, especially within restored pastures. This was mainly caused by high variation in hoverfly abundance (Supplementary information, Fig. S1 and Table S1).

### Network size

Mean network size per pasture category was 5.7 ± 1.15 plant species × 7.8 ± 2.05 pollinator species in abandoned pastures, 6.9 ± 0.98 plants × 8.7 ± 1.09 pollinators in restored pastures, and 8.8 ± 1.29 plants × 9.5 ± 1.31 pollinators in continuously grazed pastures. The number of pollinator species, number of plant species and the number of links per species in the networks did not differ among pasture categories and did not change with increasing connectivity to continuously grazed grasslands in the landscape (Table 2). Among the restored pastures, there was a positive effect of time since restoration on the number of all plant species, but no effect of connectivity on the number of all plants, pollinators or interaction links (Table 3).

**Table 2:** Summary of likelihood ratio tests, assessing the effect of ‘Connectivity’ and ‘Pasture category’ on the number of pollinator species, the number of plant species and the number of links per species in plant-pollinator networks within abandoned, restored and continuously grazed semi-natural pastures (N = 38).

	No. of pollinators			No. of plants			No. of links per species		
	χ^2^	d.f.	p	χ^2^	d.f.	p	χ^2^	d.f.	p
Connectivity	0.28	1	0.60	0.01	1	0.93	0.018	1	0.50
Pasture category	0.64	2	0.73	2.84	2	0.24	0.002	2	0.98
	**Est**.	**SE**	**p**	**Est**.	**SE**	**p**	**Est**.	**SE**	**p**
Connectivity	0.007	0.01	0.60	−0.001	0.007	0.93	−0.005	0.007	0.50
Continuous	0.21	0.26	0.42	0.25	0.15	0.09	0.03	0.14	0.83
Restored	0.12	0.23	0.62	0.16	0.13	0.22	0.01	0.12	0.92

**Table 3:** Summary of generalized linear models for the number of pollinator species, the number of plant species and the number of links per species in the plant-pollinator networks in restored semi-natural pastures (N = 18), testing for the effect of ‘Connectivity’ and ‘Time since restoration’.

	No. of pollinators			No. of plants			No. of links per species		
	Estimate	SE	p	Estimate	SE	p	Estimate	SE	p
Connectivity	−0.007	0.016	0.67	−0.005	0.010	0.63	0.003	0.008	0.72
Time since restoration	−0.043	0.031	0.17	0.038	0.018	**0**.**04**	0.024	0.017	0.16

### Beta diversity of species and interactions

#### Pairwise comparison of networks

The average beta diversity was high for both plants (β_L_, mean = 0.74) and pollinators (β_U,_ mean = 0.81), indicating a high turnover of species among networks. Beta diversity of plant (β_L_) species in interaction networks differ between pasture categories (ADONIS_,_ F_2.35_ = 1.33, R^2^ = 0.07, P = 0.02; Fig. 2). Similarly, the beta diversity of pollinators (β_U_) was dependent on pasture category (F_2.35_ = 1.65, R^2^ = 0.09, P = 0.001, Supplementary information, Table S1). The beta diversity among pasture categories resulted not only from differences in abundance, but also from changes in species composition. This is revealed from the analyses of binary networks, where the composition of both plants (F_2.35_ = 1.86, R^2^ = 0.1, P = 0.001) and pollinators (F_2.35_ = 1.83, R^2^ = 0.1, P = 0.001) differed among pasture categories.

The plant species NMDS plot revealed that restored sites partly overlapped with continuous ones and were located in-between abandoned and continuously managed sites, and abandoned sites were clearly separated from other site types (Fig. 2a). The pattern is less well visible in the pollinator species NMDS (Fig. 2b). However, separate NMDS graphs for Diptera and Hymenoptera show a clearer pattern (Supplementary information, Fig. S2): for hoverflies and bees restored sites overlap well with continuous ones but not with abandoned, thus again indicating that restoration give communities of these insect groups that resemble those in continuously grazed pastures, which is what the restoration was aiming to.

The beta diversity of pollinators was explained both by the networks having divergent sets of species involved in the interactions (β_U3_ explained 52% of β_U_ across all networks), and differences in pollinator species number (β_Urich_ explained 48%). Plant beta diversity in networks was explained equally by β_L3_ and β_Urich_ (each 50% of β_L_ each, across all networks).

The average beta diversity of interactions (β_WN_) was very high, and despite a high variability within all three pasture categories, the beta diversity of interactions tended to differ among pasture categories (β_WN_, mean = 0.99, F_2.35_ = 1.05, R^2^ = 0.06, P = 0.07) (Fig. 2c). Interaction beta diversity (β_WN_) was driven by the turnover of links (β_WN3_ explained 55% of β_WN_ across all networks), and to a lesser extent by the number of links within networks (45% explained by β_WNrich_). The average beta diversity for interactions (β_OS_) when taking into account only shared species between networks was 66%. This means that turnover of interactions between co-occurring species among networks was relatively common, but it could not be explained by pasture management category (F_2.35_ = 1.98, R^2^ = 0.10, P = 0.22). This turnover of interactions (β_OS_) was attributable mainly to differences in the number of links (β_OSrich_, 72%) and to a lesser extent to species rewiring (β_OS3_, 28%). Thus, only a small fraction (β_OS_ * β_OS3_ = 0.66 * 0.28 = ~17%) of interactions are truly rewired among networks, but this level of rewiring is still relatively high in comparison to the previous findings^16^. In summary, the turnover of both species and links was strikingly high among networks, and co-occurring species displayed relatively high interaction rewiring.

#### Local network similarity to the metaweb

When comparing each local network to the metaweb, i.e. the aggregated network including all interactions observed across all sites, the average beta diversity of interactions of each realization to the metaweb (β_OS’_) was 0.41, indicating that more than half of the potential interactions were not realized. The similarity to the metaweb in terms of interactions among shared species was not influenced by connectivity, but continuously grazed pastures have lower number of realized links than abandoned and restored pastures (Table 4). Connectivity and time since restoration had no effect on local restored network similarity to the potential network structure observed across all sites (Table 4).

**Table 4:** Summary of models on similarity of plant-pollinator networks compared to the metaweb in terms of overall interactions among shared species (OS’), testing for (i) the effect of ‘Pasture category’ and ‘Connectivity’ on network similarity for all pastures (N = 38, reference pasture state: Continuously grazed), and (ii) the effect of ‘Time since restoration’ and ‘Connectivity’ on network similarity for restored pastures (N = 18).

	OS’: all pastures included				OS’ restored pastures only		
	Estimate	SE	p		Estimate	SE	p
Connectivity	0.009	0.030	0.78	Connectivity	0.041	0.038	0.29
Pasture state: Abandoned	−1.225	0.552	**0**.**03**	Time since restoration	−0.103	0.064	0.11
Pasture state: Restored	−1.090	0.485	**0**.**03**				

## Discussion

Decomposition of interaction beta diversity revealed that plant-pollinator networks recover after restoration of semi-natural grasslands. Indeed, species interactions in previously abandoned pastures were more similar to continuously grazed pastures after restoration. Further, connectivity had no effect on species or links within networks which suggests that plant-pollinator interactions can be restored even in relatively isolated grassland patches (mean distance to continuously grazed sites was 238.12 m, SE = ±3.33). Such successful network restoration likely results from high behavioural plasticity of pollinators which is inferred from the ability of pollinators to establish interactions with the available flowering plants and a relatively high rewiring level. Indeed, most observed pollinator species were not strict diet specialists. In addition, restored grasslands hosted a high diversity of pollinators. Plant diversity was similar across pasture categories, but restored site plant composition (revealed by NMDS, Fig. 2a) was more similar to that of continuously grazed than abandoned sites. Finally, we found that abandoned grasslands were important for maintaining biodiversity as they supported a distinctive set of species and interactions.

The turnover of species among pasture categories was high both for flowering plants and pollinators, which resulted in high turnover of interactions. As well, we observed changes in interactions among co-occurring species (β_OS_, 66%) which were at the very similar level of those found by^17^. Importantly, most of the observed beta diversity (β_OS_) was not due to species rewiring (β_OS3_, 28%), but was instead mainly due to a reduction in diet breadth, i.e. differences in the number of links (β_OSrich_, 72%). Thus, only a small fraction (~17%) of interactions were truly rewired between networks. However, this demonstrates relatively high host switch level in comparison with the levels detected previously. For example, Simanonok and Burkle 2014^16^ found that host switching accounted only for 5% of interaction turnover. This demonstrates that partitioning of β_OS_ into its components (β_OS3_ and β_OSrich_) is necessary to reveal true species rewiring within networks. Such partitioning is important for biodiversity conservation purposes as it reveals whether present species in networks are able to adapt to environmental changes. Indeed, the high β_OSrich_ and relatively high pollinator host switch level (=species rewiring, β_OS3_) that we observed indicate that pollinators were able to respond to environmental changes through shifts in resource use behaviour^53^. This pollinator plasticity in resource use suggests that a restored pollinator interaction networks can be expected if the species are only able to colonize. To some extent, the high turnover of interactions observed between sites could be due to insufficient sampling, such that many of the rare interaction links in a given site are undetected. However, by using a quantitative approach, and not only analysing presence-absence of links, we are minimizing this effect.

The different sources of interaction beta diversity show that plant-pollinator networks are highly plastic. This could buffer the impact of species loss^54^. It has been reported that plant-pollinator interactions can vary over time depending on the presence and abundance of plant species, even for specialised pollinators^55,56^. As a result, species abundance declines or even species extinctions related to grassland management changes do not necessarily cause secondary extinctions. Instead, at least some interacting species can persist through behavioural changes. Such behavioural plasticity is especially important in keeping ecosystem stability after restoration actions and could facilitate network integrity^7^ and adaptation to invasive species^57^. Also, this could help to buffer phenological mismatch of interacting species due to climate change^58,59^. Generally, behavioural plasticity in networks is critically important in maintaining ecosystem resilience and resistance to environmental change and on the stable provision of wild plant pollination.

Plant and pollinator species composition in networks (beta-diversity) were different depending on the pasture category. This means that management affects communities which increases beta-diversity within landscapes. It is well recognized that intact semi-natural grasslands are key habitats for biodiversity in the agricultural landscape^60^, but our results indicate that the presence of degraded and newly restored grasslands increase the regional species pool. Indeed, abandoned sites hosted very different plant communities (Fig. 2a), as well as distinctive set of pollinator species and interactions. Restored sites most profoundly differed from other sites in having highest pollinator richness and abundance. Apparently, recently restored pastures have qualities which are important for pollinators and are lacking from both abandoned and continuously grazed grasslands. Since there was no difference in the abundance of floral resources among pasture categories, we suggest that recently restored sites likely provide more bee nesting sites which might often be a limiting resource^34,61,62^. Indeed, recently restored sites typically contain patches of bare ground that may provide nesting sites for ground-nesting bees. 36 of the 63 non-parasitic bee species observed in restored pastures depend on nesting sites in the ground. This is supported by the observation that the overlap in community composition between restored and both abandoned and continuously grazed pastures was relatively small, i.e. the restored sites contain a distinct set of species that do not occur in any of the other habitat categories. We propose that as long as abandoned grasslands are being restored before succession has progressed too far^63,64^, and as long as there are intact grasslands within the landscape^65^, temporary local grassland abandonment does not have to be negative. Instead, differences in successional stages of semi-natural grasslands within the landscape can support a high variety of species and interactions, and contribute to the overall diversity and functioning of the agricultural landscape.

Contrary to our expectations, species or links within networks, as well as the proportion of realized links in a site (i.e. with compared to potential links in the metaweb) was not affected by connectivity or the time since restoration. This could potentially be due to the choice of connectivity measure. However, we have previously observed, in the same landscapes, that the composition of pollinator communities is structured by the connectivity to other continuously grazed grasslands^32,34^ even though the number of species is the same. This effect of connectivity on species composition could at least partly explain the observed high turnover of pollinator species among sites. Instead, the lack of effects of connectivity highlights the importance of local habitat factors in link realization and overall success of plant-pollinator network restoration. The local plant community is an important determinant of pollinator communities^35^, but pollinators are highly mobile organisms, making rapid recovery of pollinator communities and plant-pollinator interaction networks possible after restoration, even in relatively isolated grasslands. The rapid recovery of interaction networks is also made possible by the observed high turnover of co-occurring species and relatively high degree of rewiring, indicating a behavioural plasticity in terms of shifting diets. Pollinators are plastic at two levels: (i) using novel partners when the preferred ones are not available, (ii) using different partners even when preferred partners are available. Such behavioural plasticity is especially important in maintaining the ability of ecosystems to counteract negative effects of human-caused environmental changes.

## Supplementary information

Plant-pollinator network restoration in grasslands

## Data Availability

We intend to archive the data of this publication in Dryad Digital Repository (https://datadryad.org/).
