# Supplementary material for: Pollinator foraging flexibility mediates rapid plant-pollinator network restoration in semi-natural grasslands
Source: Sci Rep. 2019 Oct 29;9:15473. doi: 10.1038/s41598-019-51912-4 (PMC6820780; doi:10.1038/s41598-019-51912-4)
Supplement: Supplementary file 1 — Plant-pollinator network restoration in grasslands [file 41598_2019_51912_MOESM1_ESM.docx]

# Pollinator foraging flexibility mediates rapid plant-pollinator network restoration in semi-natural grasslands

Norbertas Noreika, Ignasi Bartomeus, Marie Winsa, Riccardo Bommarco, Erik Öckinger

**Supporting information**

Figure S1. Mean a) number of hoverfly species, b) number of bee species, c) hoverfly abundance and d) bee abundance per site and pasture category ±95% confidence interval. Note that the numbers correspond to all observed pollinator species and individuals, not only the ones included in local networks.


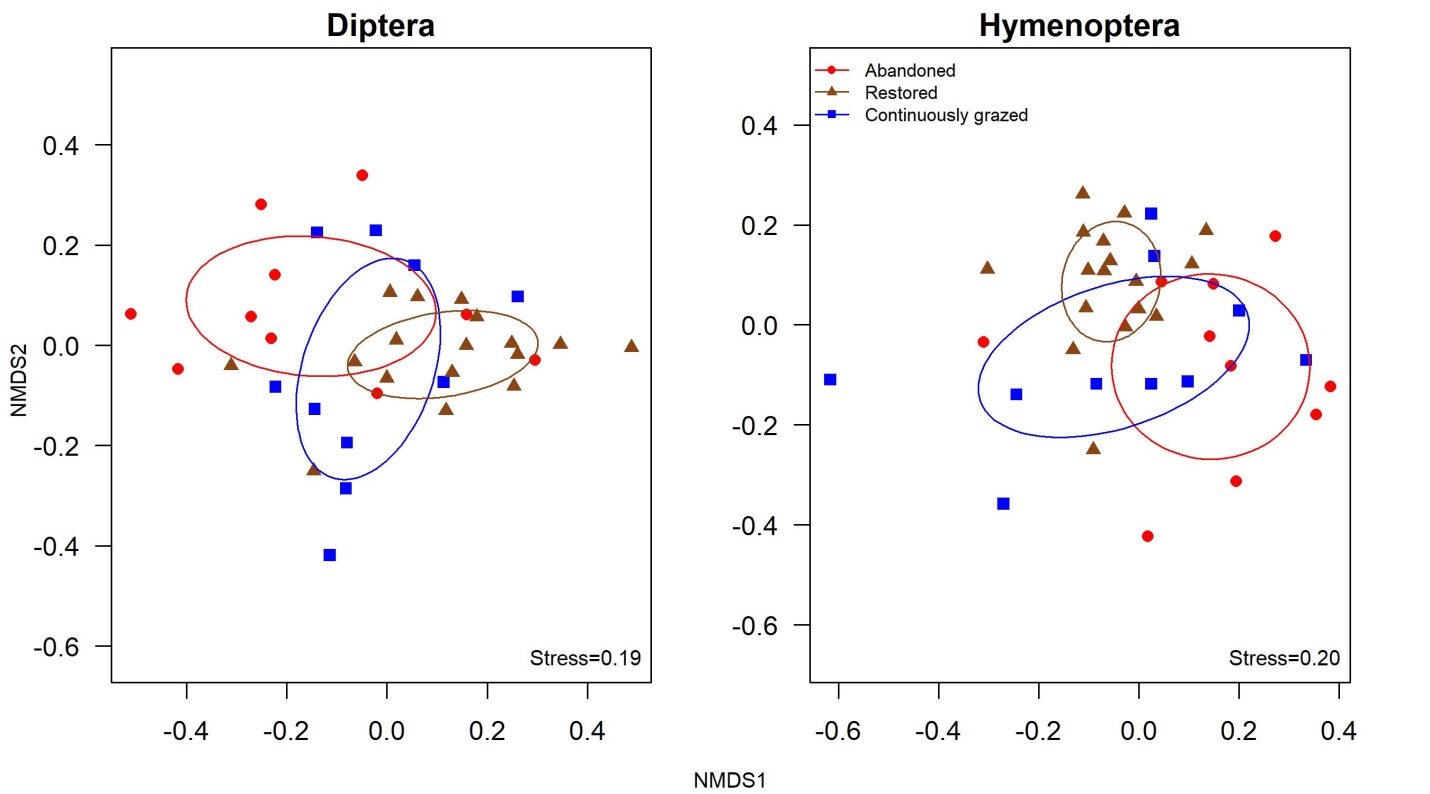


Figure S2. NMDS visualizing beta diversity of Diptera and Hymenoptera composition (without outlier) in three pasture categories.

Table S1. Pollinator and flowering plant species number and abundance per pasture, with mean and SE for each pasture category. Both overall richness/abundance, and the richness/abundance within networks are reported. Means are given separately for bees and hoverflies, but in the network analyses the two groups were pooled.

|  |  | Abandoned | |  | Restored | |  | Continuous | |
| --- | --- | --- | --- | --- | --- | --- | --- | --- | --- |
|  | Species group | Mean | SE |  | Mean | SE |  | Mean | SE |
| Number of species per site | Hoverflies | 8.6 | 2.4 |  | 15.7 | 2.0 |  | 10.6 | 1.7 |
|  | Bees | 6.9 | 1.1 |  | 16.8 | 1.9 |  | 9.0 | 2.2 |
|  | Flowering plants | 22.8 | 2.2 |  | 26.8 | 2.2 |  | 29.2 | 2.8 |
| Abundance per site | Hoverflies | 25.6 | 11.7 |  | 88.6 | 30.9 |  | 27.6 | 10.0 |
|  | Bees | 11.7 | 2.7 |  | 44.7 | 8.4 |  | 19.2 | 6.4 |
|  | Flowers/inflorescences  *(per 1m^2^)* | 15.1 | 4.2 |  | 16.7 | 2.6 |  | 24.5 | 5.1 |
| Number of species per network | Hoverflies | 4.0 | 1.6 |  | 3.7 | 0.5 |  | 4.9 | 0.8 |
|  | Bees | 3.8 | 1.6 |  | 5.0 | 0.8 |  | 4.6 | 0.7 |
|  | Flowering plants | 5.7 | 1.1 |  | 6.9 | 1.0 |  | 8.8 | 1.3 |
| Individuals in networks | Hoverflies | 11.8 | 2.5 |  | 6.1 | 1.1 |  | 8.1 | 2.5 |
|  | Bees | 5.6 | 1.4 |  | 8.3 | 2.2 |  | 8.0 | 1.8 |

Table S2. Pollinator species and total number of individuals observed from three pasture categories (abandoned, restored, continuously grazed). These include both individuals caught flying and those observed visiting flowers.

| Species | Pasture category |  |  |
| --- | --- | --- | --- |
|  | **Abandoned** | **Restored** | **Continuous** |
| **Hymenoptera** |  |  |  |
| *Andrena bicolor* | 0 | 0 | 1 |
| *Andrena carantonica* | 0 | 2 | 0 |
| *Andrena cineraria* | 0 | 2 | 1 |
| *Andrena fucata* | 5 | 2 | 0 |
| *Andrena fulvago* | 0 | 3 | 2 |
| *Andrena fuscipes* | 3 | 8 | 1 |
| *Andrena haemorrhoa* | 2 | 63 | 2 |
| *Andrena helvola* | 4 | 9 | 0 |
| *Andrena lapponica* | 0 | 1 | 0 |
| *Andrena lathyri* | 0 | 2 | 0 |
| *Andrena nigroaenea* | 1 | 3 | 0 |
| *Andrena nitida* | 1 | 0 | 0 |
| *Andrena praecox* | 0 | 1 | 0 |
| *Andrena saundersella* | 1 | 6 | 2 |
| *Andrena subopaca* | 2 | 14 | 3 |
| *Andrena wilkella* | 1 | 7 | 4 |
| *Anthidium punctatum* | 0 | 6 | 4 |
| *Anthophora furcata* | 0 | 1 | 1 |
| *Bombus barbutellus* | 0 | 2 | 2 |
| *Bombus bohemicus* | 5 | 30 | 8 |
| *Bombus campestris* | 2 | 10 | 1 |
| *Bombus hortorum* | 6 | 16 | 3 |
| *Bombus humilis* | 1 | 25 | 6 |
| *Bombus hypnorum* | 4 | 11 | 10 |
| *Bombus jonellus* | 0 | 5 | 1 |
| *Bombus lapidarius* | 6 | 75 | 10 |
| *Bombus lucorum* | 17 | 77 | 15 |
| *Bombus norvegicus* | 0 | 2 | 0 |
| *Bombus pascuorum* | 24 | 137 | 51 |
| *Bombus pratorum* | 3 | 19 | 4 |
| *Bombus quadricolor* | 0 | 0 | 1 |
| *Bombus ruderarius* | 2 | 38 | 16 |
| *Bombus rupestris* | 0 | 6 | 0 |
| *Bombus soroeensis* | 2 | 17 | 4 |
| *Bombus subterraneus* | 1 | 11 | 3 |
| *Bombus sylvarum* | 3 | 32 | 4 |
| *Bombus sylvestris* | 0 | 1 | 0 |
| *Bombus terrestris* | 4 | 38 | 10 |
| *Chelostoma florisomne* | 2 | 5 | 1 |
| *Coelioxys inermis* | 0 | 1 | 0 |
| *Colletes daviesanus* | 0 | 3 | 0 |
| *Eucera longicornis* | 0 | 10 | 3 |
| *Halictus rubicundus* | 0 | 0 | 1 |
| *Halictus tumulorum* | 1 | 15 | 2 |
| *Heriades truncorum* | 0 | 0 | 1 |
| *Hoplitis claviventris* | 0 | 2 | 1 |
| *Hoplitis leucomelana* | 0 | 1 | 0 |
| *Hylaeus communis* | 0 | 4 | 0 |
| *Hylaeus confusus* | 0 | 1 | 0 |
| *Hylaeus hyalinatus* | 4 | 2 | 0 |
| *Lasioglossum albipes* | 3 | 10 | 2 |
| *Lasioglossum calceatum* | 0 | 4 | 1 |
| *Lasioglossum fratellum* | 0 | 1 | 0 |
| *Lasioglossum fulvicorne* | 1 | 1 | 0 |
| *Lasioglossum leucopus* | 0 | 10 | 1 |
| *Lasioglossum leucozonium* | 0 | 0 | 1 |
| *Lasioglossum morio* | 0 | 1 | 0 |
| *Lasioglossum punctatissimum* | 0 | 1 | 0 |
| *Lasioglossum villosulum* | 0 | 1 | 0 |
| *Lasioglossum zonulum* | 0 | 2 | 0 |
| *Macropis europaea* | 0 | 1 | 0 |
| *Megachile alpicola* | 0 | 1 | 0 |
| *Megachile centuncularis* | 0 | 1 | 0 |
| *Megachile versicolor* | 0 | 3 | 0 |
| *Nomada flavoguttata* | 1 | 5 | 0 |
| *Nomada fulvicornis* | 0 | 2 | 0 |
| *Nomada goodeniana* | 0 | 2 | 0 |
| *Nomada lathburiana* | 1 | 4 | 0 |
| *Nomada leucophthalma* | 0 | 1 | 0 |
| *Nomada marshamella* | 0 | 1 | 0 |
| *Nomada moeschleri* | 0 | 5 | 0 |
| *Nomada ruficornis* | 0 | 7 | 2 |
| *Nomada striata* | 1 | 3 | 0 |
| *Osmia bicolor* | 2 | 0 | 2 |
| *Osmia bicornis* | 0 | 2 | 0 |
| *Osmia caerulescens* | 0 | 1 | 0 |
| *Osmia nigriventris* | 0 | 1 | 0 |
| *Osmia pilicornis* | 0 | 3 | 0 |
| *Sphecodes crassus* | 0 | 1 | 1 |
| *Trachusa byssina* | 1 | 3 | 3 |
| **Diptera** |  |  |  |
| *Anasimyia interpuncta* | 0 | 1 | 0 |
| *Brachypalpoides lentus* | 0 | 2 | 0 |
| *Cheilosia albitarsis* | 0 | 9 | 2 |
| *Cheilosia fraterna* | 0 | 1 | 0 |
| *Cheilosia latifrons* | 0 | 1 | 0 |
| *Cheilosia nebulosa* | 0 | 1 | 0 |
| *Cheilosia pagana* | 1 | 2 | 3 |
| *Cheilosia psilophthalma* | 0 | 1 | 0 |
| *Cheilosia scutellata* | 2 | 0 | 0 |
| *Cheilosia urbana* | 0 | 1 | 1 |
| *Cheilosia uviformis* | 0 | 0 | 1 |
| *Cheilosia velutina* | 0 | 2 | 0 |
| *Cheilosia vernalis* | 0 | 1 | 1 |
| *Cheilosia vicina* | 0 | 3 | 1 |
| *Chrysogaster solstitialis* | 0 | 3 | 1 |
| *Chrysotoxum arcuatum* | 1 | 1 | 0 |
| *Chrysotoxum bicinctum* | 2 | 9 | 0 |
| *Chrysotoxum festivum* | 0 | 1 | 1 |
| *Dasysyrphus hilaris* | 1 | 4 | 0 |
| *Dasysyrphus pinastri* | 1 | 2 | 2 |
| *Dasysyrphus tricinctus* | 4 | 3 | 0 |
| *Dasysyrphus venustus* | 2 | 6 | 2 |
| *Didea alneti* | 3 | 0 | 0 |
| *Didea fasciata* | 1 | 1 | 0 |
| *Didea intermedia* | 2 | 1 | 1 |
| *Doros profuges* | 0 | 1 | 0 |
| *Epistrophe eligans* | 0 | 9 | 0 |
| *Epistrophe flava* | 1 | 0 | 0 |
| *Epistrophe grossulariarae* | 1 | 0 | 0 |
| *Epistrophe nitidicollis* | 1 | 0 | 0 |
| *Epistrophe obscuripes* | 0 | 1 | 0 |
| *Episyrphus balteatus* | 50 | 167 | 14 |
| *Eristalinus sepulchralis* | 0 | 1 | 0 |
| *Eristalis arbustorum* | 0 | 54 | 1 |
| *Eristalis interrupta* | 18 | 177 | 45 |
| *Eristalis lineata* | 0 | 105 | 10 |
| *Eristalis pertinax* | 0 | 0 | 2 |
| *Eristalis picea* | 0 | 61 | 3 |
| *Eristalis pseudorupium* | 2 | 51 | 4 |
| *Eristalis rupium* | 1 | 50 | 1 |
| *Eristalis tenax* | 0 | 1 | 0 |
| *Eumerus strigatus* | 0 | 0 | 2 |
| *Eupeodes bucculatus* | 0 | 1 | 1 |
| *Eupeodes corollae* | 2 | 8 | 2 |
| *Eupeodes luniger* | 0 | 1 | 0 |
| *Ferdinandea cuprea* | 0 | 1 | 0 |
| *Ferdinandea ruficornis* | 0 | 2 | 0 |
| *Helophilus affinis* | 0 | 2 | 2 |
| *Helophilus hybridus* | 1 | 6 | 2 |
| *Helophilus pendulus* | 10 | 90 | 20 |
| *Helophilus trivittatus* | 0 | 1 | 0 |
| *Megasyrphus erraticus* | 4 | 8 | 1 |
| *Melangyna umbellatarum* | 5 | 0 | 0 |
| *Melanogaster aerosa* | 0 | 2 | 0 |
| *Melanostoma mellinum* | 13 | 66 | 23 |
| *Melanostoma scalare* | 3 | 3 | 0 |
| *Meliscaeva cinctella* | 2 | 2 | 2 |
| *Microdon analis* | 0 | 1 | 0 |
| *Microdon mutabilis* | 0 | 1 | 0 |
| *Myathropa florea* | 1 | 2 | 0 |
| *Neoascia geniculata* | 0 | 2 | 0 |
| *Paragus haemorrhous* | 0 | 0 | 1 |
| *Paragus pecchiolii* | 0 | 0 | 1 |
| *Parasyrphus annulatus* | 0 | 1 | 1 |
| *Parasyrphus lineolus* | 3 | 4 | 0 |
| *Parasyrphus vittiger* | 0 | 1 | 0 |
| *Parhelophilus consimilis* | 0 | 1 | 0 |
| *Parhelophilus frutetorum* | 0 | 1 | 0 |
| *Pipiza austriaca* | 1 | 1 | 0 |
| *Pipiza quadrimaculata* | 2 | 0 | 0 |
| *Pipizella viduata* | 1 | 3 | 6 |
| *Platycheirus albimanus* | 1 | 5 | 0 |
| *Platycheirus clypeatus* | 0 | 23 | 3 |
| *Platycheirus fulviventris* | 0 | 2 | 0 |
| *Platycheirus granditarsis* | 0 | 1 | 0 |
| *Platycheirus rosarum* | 0 | 1 | 2 |
| *Rhingia campestris* | 0 | 9 | 2 |
| *Scaeva selenitica* | 0 | 1 | 0 |
| *Sericomyia lappona* | 0 | 0 | 1 |
| *Sericomyia silentis* | 0 | 15 | 3 |
| *Sphaerophoria batava* | 6 | 4 | 0 |
| *Sphaerophoria interrupta* | 0 | 1 | 0 |
| *Sphaerophoria philantha* | 1 | 0 | 1 |
| *Sphaerophoria scripta* | 19 | 76 | 31 |
| *Sphaerophoria taeniata* | 16 | 45 | 11 |
| *Sphaerophoria virgata* | 4 | 0 | 1 |
| *Syritta pipiens* | 0 | 20 | 1 |
| *Syrphus admirandus* | 0 | 1 | 0 |
| *Syrphus ribesii* | 27 | 293 | 34 |
| *Syrphus torvus* | 4 | 28 | 6 |
| *Syrphus vitripennis* | 33 | 102 | 16 |
| *Tropidia scita* | 0 | 0 | 1 |
| *Volucella bombylans* | 1 | 7 | 2 |
| *Volucella pellucens* | 1 | 2 | 0 |
| *Xanthogramma citrofasciatum* | 0 | 1 | 0 |
| *Xanthogramma pedissequum* | 0 | 1 | 0 |
| *Xanthogramma stackelbergi* | 1 | 1 | 0 |
| *Xylota florum* | 0 | 0 | 1 |
| *Xylota jakutorum* | 0 | 1 | 0 |
| *Xylota segnis* | 0 | 3 | 0 |
| *Xylota tarda* | 0 | 2 | 0 |
| Syrphidae 1* | 0 | 2 | 0 |
| Syrphidae 2* | 0 | 1 | 0 |
| Syrphidae 3* | 0 | 1 | 0 |
|  |  |  |  |

*- individuals not identified to species level

Table S3. Summary of beta diversity analysis from pairwise comparison of binary networks, exploring differences in network composition among pasture categories in terms of species (U: pollinators, L: plants), interactions (WN), and interactions among shared species (OS). Beta diversity metrics for pollinator composition (β_U_, P=0.10) and interactions (β_WN_, P=0.05) were further partitioned to explore if effects (or possible effects) of pasture category were due to turnover of species/interactions (U3, WN3), or differences in number of species/links (Urich, WNrich).

|  | β_U_ | β_U3_ | β_Urich_ | β_L_ | β_WN_ | β_WN3_ | β_WNrich_ | β_OS_ |
| --- | --- | --- | --- | --- | --- | --- | --- | --- |
| P | **0.10** | **0.06** | 0.40 | 0.54 | **0.05** | 0.25 | 0.38 | 0.15 |
| F | 1.21 | 1.87 | 1.03 | 0.97 | 1.07 | 1.38 | 1.03 | 2.37 |
| R^2^ | 0.06 | 0.10 | 0.06 | 0.05 | 0.06 | 0.07 | 0.06 | 0.12 |
